# Chronic restraint stress impairs cognition via modulating HDAC2 expression

**DOI:** 10.1515/tnsci-2020-0168

**Published:** 2021-04-29

**Authors:** Jie Wu, Cui Liu, Ling Zhang, Bing He, Wei-Ping Shi, Hai-Lei Shi, Chuan Qin

**Affiliations:** Pathology Department, Comparative Medical Center, Peking Union Medical College (PUMC) and Institute of Laboratory Animal Science, Chinese Academy of Medical Science (CAMS), Panjiayuan Nanli No. 5, Beijing, 100021, People’s Republic of China; Department of Pathology, Affiliated Hospital of Qingdao University, No. 16, Jiangsu Road, Qingdao, Shandong province, 266003, People’s Republic of China; Comparative Medical Center, Peking Union Medical College (PUMC) and Institute of Laboratory Animal Science, Chinese Academy of Medical Science (CAMS), Panjiayuan Nanli No. 5, Beijing, 100021, People’s Republic of China

**Keywords:** Alzheimer’s disease, chronic stress, cognition, epigenetics, histone deacetylase-2

## Abstract

**Background:**

To investigate the effects of chronic restraint stress on cognition and the probable molecular mechanism in mice.

**Methods:**

In the current work, a restraining tube was used as a way to induce chronic stress in mice. The protein levels were determined with ELISA and western blot. A series of behavior tests, including the Morris water maze, elevated plus maze, open field test, and novel object recognition test, were also performed to examine the anxiety and the ability of learning and memory. Moreover, murine neuroblastoma N2a cells were used to confirm the findings from mice under chronic stress.

**Results:**

Decreased synaptic functions were impaired in chronic stress with the downregulation of PSD95, GluR-1, the neurotrophic factor BDNF, and immediate-onset genes Arc and Egr. Chronic restraint decreased the histone acetylation level in hippocampal neurons while HDAC2 was increased and was co-localized with glucocorticoid receptors. Moreover, chronic stress inhibited the PI3K/AKT signaling pathway and induced energy metabolism dysfunctions.

**Conclusion:**

This work examining the elevated levels of HDAC2 in the hippocampus may provide new insights and targets for drug development for treating many neurodegenerative diseases.

## 1 Introduction

In modern society, with the accelerating pace of life, chronic stress has become a common problem that cannot be ignored and has become an important risk factor for the onset of many diseases including Alzheimer’s disease (AD) [1,2,3]. Two important stress response systems include the hypothalamic-pituitary-adrenal axis (HPA axis) and the sympathetic nervous system [4,5]. The sympathetic nervous system is involved in the acute stress response, whereas for chronic stress stimuli, the HPA axis is activated with increased glucocorticoid released [6,7]. Cortisol is the predominant glucocorticoids produced in mammals and exerts widespread actions in the body as a central component of the stress response [8]. Chronic stress responses are systemic reactions involving multiple organs and can induce symptoms such as depression, cognitive impairment, cardiovascular disease, immune dysfunctions, impaired reproductive ability, bone loss, and reduced life expectancy [9]. Among these risk factors, cognitive impairment is the most noticeable, as is evident in AD, but the mechanism is still unknown.

In many previous studies, researchers have been trying to elucidate the mechanisms involved in cognitive impairment under chronic stress [10,11]. Epigenetic regulation, especially the regulation of histone acetylation by histone deacetylases (HDACs), has attracted much attention to resolve this problem [12]. Histone deacetylation induces gene silencing and the downregulation of target protein expression. Previous findings have revealed that several HDAC family members that included HDAC4 and HDAC1 are involved in neuron injury caused by chronic stress [13]. However, how those HDAC members regulate memory-associated genes and mediate memory impairment are still unclear. HDACs are key enzymes that downregulate the level of histone acetylation [14] and whether chronic stress exerts cognitive damage through direct HDAC regulation has yet to be determined.

In this study, we aimed to explore the effects of chronic stress on learning and memory and further reveal the role of HDAC2 on memory-associated gene regulation.

## 2 Methods and materials

### 2.1 Animals

In this study, 3 months old (young group) and 12 months old (old age group) C57Bl/6 J mice were used. Only female mice were used and tested at same time of the day in this study to eliminate variability in results that can be caused by fluctuating levels of sex steroids and the difference in hormone levels between male and female mice.


**Ethical approval:** All animals in this program were used in strict accordance with the NIH Laboratory Animal Welfare and Ethics Regulations. All procedures were approved by Institute of Laboratory Animals Science, CAMS & PUMC.

### 2.2 Chronic restraint stress

In this experiment, a restraining tube was used as a way to induce chronic stress in mice [5]. All procedures involving animals were reviewed and approved by Institute of Laboratory Animals Science for ensuring animal care. To induce chronic restraint stress, the mice were placed in restraint tube to restrict its activity. The restraint tube was a cylindrical plastic sleeve with a diameter of 3–4 cm and the length is adjustable. The tube wall was transparent and had a small ventilation port on one side. Therefore, when the mice were placed in the restraint tube, its physical activities were limited but not its breathing. The mice were restrained in the tube for 8 h (11:00 p.m. to 7:00 a.m.) per day for 29 days. When not in the restraining tube, the mice were placed in a cage with free access to food and water.

### 2.3 Open field test

The open field test (OFT) is a well-established test for determining the anxiety and stress levels of small rodents. The OFT is performed in an open rectangular box with a length of 50 cm, a width of 50 cm, and a height of 40 cm. The footprint was divided into three different parts: the center zone, border zone, and transfer zone. During the experiment, the mice were placed in the open field at the same position facing the side wall. The spontaneous activities of mice in the open field for a duration of 5 min were recorded by the Ethovision XT monitoring and analysis system. The time spent in the three zones was later analyzed from this recording. 70% ethanol solution was used to clean the box between animals.

### 2.4 Elevated plus maze

The elevated plus maze (EPM) is another behavioral measure suitable for evaluating anxiety states in mice. The physical device is comprised of four arms, two closed arms (length 30 cm, width 5 cm, and depth 5 cm) and two open arms (length 30 cm and width 5 cm). The central area (5 cm long and 5 cm wide) forms the center intersection of the two closed arms. During this test, the mice were placed in the central area and were allowed to move spontaneously among the four arms for 5 min. The activity of the mice was recorded by the Ethovision XT monitoring and analysis system. The frequency and time spent in each arm (closed vs open) were later analyzed. The anxiety index (AI) was calculated according to equation (1) where {t}_{\text{open}\text{arm}}] and {t}_{\text{closed}\text{arm}}] represent the time spent in the open and closed arms, respectively, and where {\#}_{\text{open}\text{arm}}] and {\#}_{\text{closed}\text{arm}}] represent the number of times entering the open and closed arms, respectively.

(1) \text{AI}=\text{ }\frac{\frac{{t}_{\text{open}\text{arm}}}{{t}_{\text{open}\text{arm}}+{t}_{\text{closed}\text{arm}}}}{\frac{{\#}_{\text{open arm}}}{{\#}_{\text{open}\text{arm}}+{\#}_{\text{closed}\text{arm}}}}\times 100.]

### 2.5 Morris water maze

The Morris water maze is a classic paradigm for learning and memory-related behavioral evaluations. This device consisted of a circular pool with a 2 m diameter and a video recording system [15]. The pool was divided into four quadrants and a platform was placed in the center of one quadrant, known as the target quadrant. The position of the platform remained unchanged during the test. On day 0, the platform was visible and the mice were guided to the platform for 15 s. On days 1–5, the platform was hidden and the mouse being tested was required to swim and find the location of the platform placed below the surface of water in 60 s. Each mouse underwent four training trials per day from the different quadrants. On the sixth day, the probe test was conducted to measure spatial bias with the platform removal. The swimming activity of each mouse was monitored by an overhead video camera and analyzed using Noldus Ethovision XT software.

### 2.6 Novel object recognition

The novel object recognition test is another experimental measure for evaluating cognitive functions mainly involving short-term memory [16]. The experimental device consisted of a plastic box 45 cm in length, 30 cm in width, and 20 cm in height. On the first and second days, the mouse was placed in the box and habituated to the open area for 5 min/day. On the third and fourth days, in the training phase, two identical objects and the mouse were placed in the box and allowed to explore for 5 min, after which the mice were returned to their home cage. During the test phase on day 5, one of the two objects was replaced with a novel object in the same position, and the mouse was placed in the box and allowed to explore for 5 min. The exploration time for the new (TN) and old/familiar (TF) objects during the 5 min evaluation was recorded. The discrimination index (DI) was calculated according to equation (2):

(2) \text{DI}=\hspace{.25em}\frac{\text{TN}-\text{TF}}{\text{TN}+\text{TF}}\times 100.]

### 2.7 Protein extraction and western blot assay

The mice were killed after anesthesia and the cortex and hippocampus were isolated. One milliliter of lysis buffer was added for every 0.1 g of brain tissue to a sterilized container. The mixture was homogenized, lysed on ice for 30 min, and then centrifuged at 4°C at 13,000 rpm for 30 min. The supernatant was harvested and stored at −80°C. The protein concentration was determined using the BCA Protein Assay (ThermoFisher, USA) according to the manufacturer’s instruction. Total protein samples (30 μg) were loaded on SDS-PAGE and then transferred onto PVDF membranes. The membranes were blocked with 5% BSA for 1 h at room temperature (RT) and incubated with primary antibodies at 4°C overnight. The membranes were then washed with PBST and incubated with the secondary antibodies for 1 h at RT. Proteins were visualized using ECL solution and the intensity values were analyzed using the ImageJ software.

### 2.8 ELISA of serum corticosteroid levels

At the end of all behavioral tests, the blood of mice was collected. The blood samples were placed at RT until they were completely coagulated. The samples were then centrifuged at 4°C at 5,000 rpm for 20 min to obtain the supernatant and stored at −80°C. The serum corticosteroid levels were determined using the ELISA kit according to the manufacturer’s instructions.

### 2.9 Immunofluorescence

The mice brains were fixed in 4% paraformaldehyde and cut into sections using a cryostat, and stored at −20°C for later use. The sections were rinsed thrice with PBS for 2 min, supplemented with 0.5% Triton buffer for 5 min. The sections were then washed thrice with PBS for 2 min and incubated with 3% H_2_O_2_ for 10 min. The endogenous peroxidase was blocked and slices were washed thrice with PBS for 2 min. After blocking for 20 min with 5% BSA, the primary antibody was added and incubated at 4°C overnight. On the next day, the sections were washed thrice and labeled with a secondary antibody and incubated for 20 min in the dark room. After washing thrice with PBS, the slides were sealed with a DAPI mounting solution. The images were captured by fluorescent microscopy, and the fluorescence density was analyzed using the Image J software.

### 2.10 Cell culture and transfection

Murine neuroblastoma N2a cells were maintained at 37°C in a 5% CO_2_ incubator in DMEM with 10% fetal bovine serum and penicillin/streptomycin (100 μg/mL). N2a cells were transfected with mouse HDAC short hairpin RNA (shRNA) and scrambled shRNA using Lipofectamine 3000 Reagent from Invitrogen. After 24–72 h post-transfection, cells were used for analysis. HDAC shRNA: 5′-TACGATACAAGGCTGTTAGAGAG-3′.

### 2.11 Statistical analysis

Statistical analysis was performed using the software SPSS 19.0. Data are presented as the mean ± SE. For comparing two groups, Student’s *t*-test was used. For multi-group comparison, one-way analysis of variance (ANOVA) with the least-significant difference (LSD) *post hoc* test was used. The hidden platform test results of the Morris water maze were analyzed using two-way ANOVA. *p* < 0.05 was considered statistically significant.

## 3 Results

### 3.1 Chronic restraint stress impaired cognition in mice

**Figure 1:**
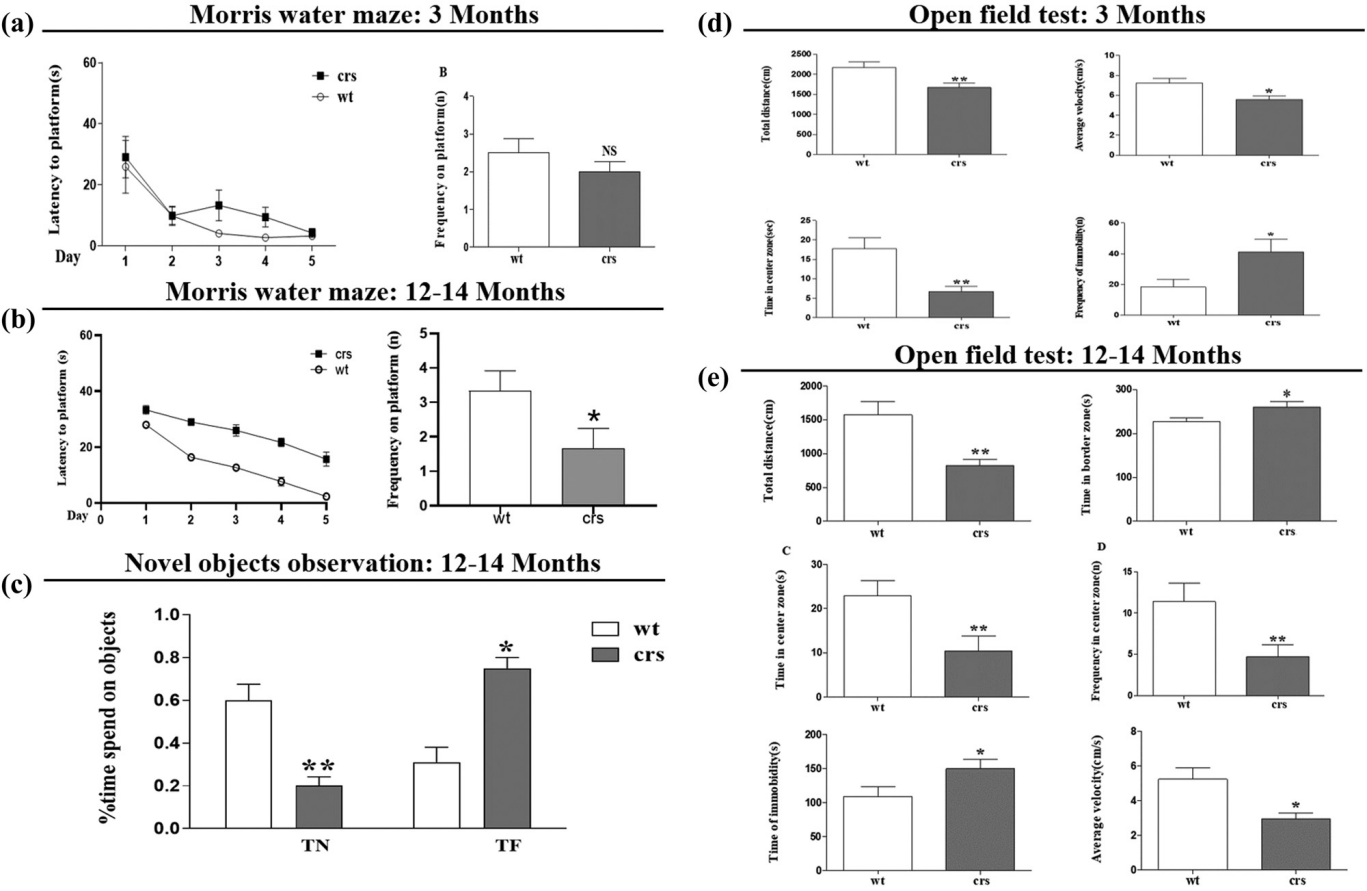
Chronic restraint stress impaired cognition in mice. (a) Latency for reaching the platform and frequency of platform crossings in the Morris water maze test of 3-month-old mice. (b) Latency for reaching the platform and frequency of platform crossings in the Morris water maze test of 12-month-old mice. Frequency on platform: *p = 0.039. (c) The results of the novel object recognition test of 12-month-old mice, **p = 0.009, *p = 0.021. (d) The total distance traveled in the open field, the cumulative time spent in the border and center areas, the time of center area crossings, the frequency of immobility, and average velocity in the OFT of 3-month-old mice. Total distance: **p = 0.0090; average velocity: *p = 0.0110; time in center zone: **p = 0.0072; frequency of immobility: *p = 0.0024. (e) The total distance traveled in the open field (**p = 0.0040), cumulative time spent in the border (**p = 0.0406) and center areas (**p = 0.0098), frequency of center area crossings (**p = 0.0021), time of immobility (**p = 0.0466), and average velocity (**p = 0.0075) in the open filed test of 12-month-old mice. *N* = 10–12 per group; *p < 0.05; **p < 0.01.

To explore the effects of chronic stress on learning and memory, mice of different ages (3 and 12 months) were used in chronic restraint stress experiments. Behavioral tests include the Morris water maze (Figure 1a and b), novel objects recognition tests (Figure 1c), and the OFT (Figure 1d). The results showed that chronic restraint stress impaired the learning and memory abilities in mice, but that the effects varied among mice of different ages. In young mice of 3 months, depression and anxiety-like behaviors were apparent under chronic stress. However, in older mice under chronic stress, the changes in cognitive behaviors became more apparent. To reveal the mechanism of stress-induced memory impairment, we used aged mice of 12 months in the following experiments.

### 3.2 Chronic restraint stress decreased synaptic proteins

**Figure 2:**
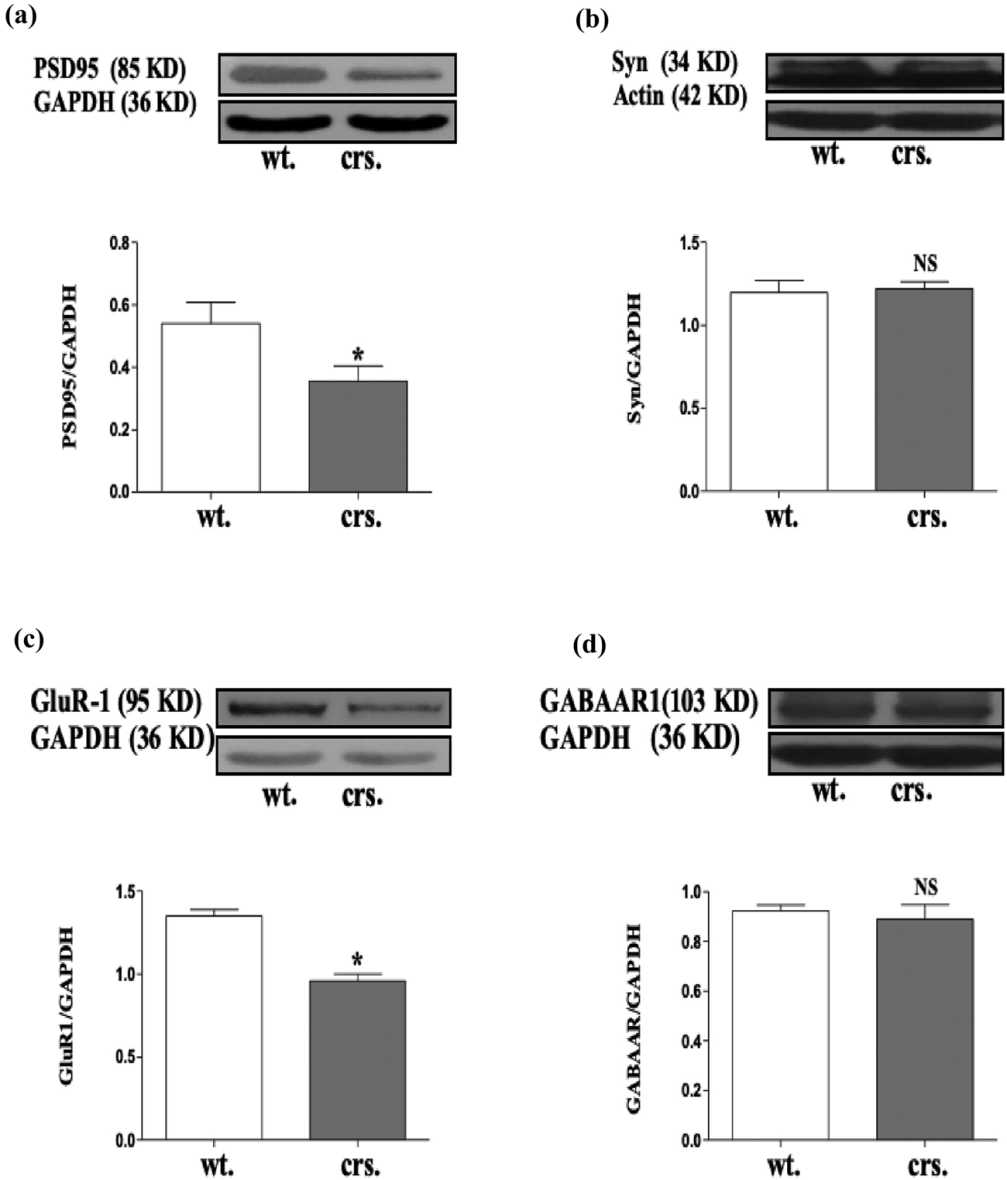
Protein levels of PSD95 (a), Syn (b), GluR-1 (c), and GABAAR (d) after the induction of chronic stress. *N* = 3, (a), *p = 0.035; (c), *p = 0.0480; NS, not significant.

Synapse-associated proteins are important for maintaining long-term potentiation (LTP) and cognitive functions including learning and memory. To investigate the reason of stress-induced memory impairment, we examined changes in proteins associated with synaptic functions [17]. Western blot analysis showed that the expression of post-synaptic PSD95 (*p* = 0.031) and the glutamate receptor GluR-1 (*p* = 0.029) were both significantly decreased in chronically stressed animals compared with the control group (Figure 2). These results suggest that the expression levels of synaptic proteins were impaired in chronic stress.

### 3.3 Higher serum corticosteroid was observed after chronic restraint stress

**Figure 3:**
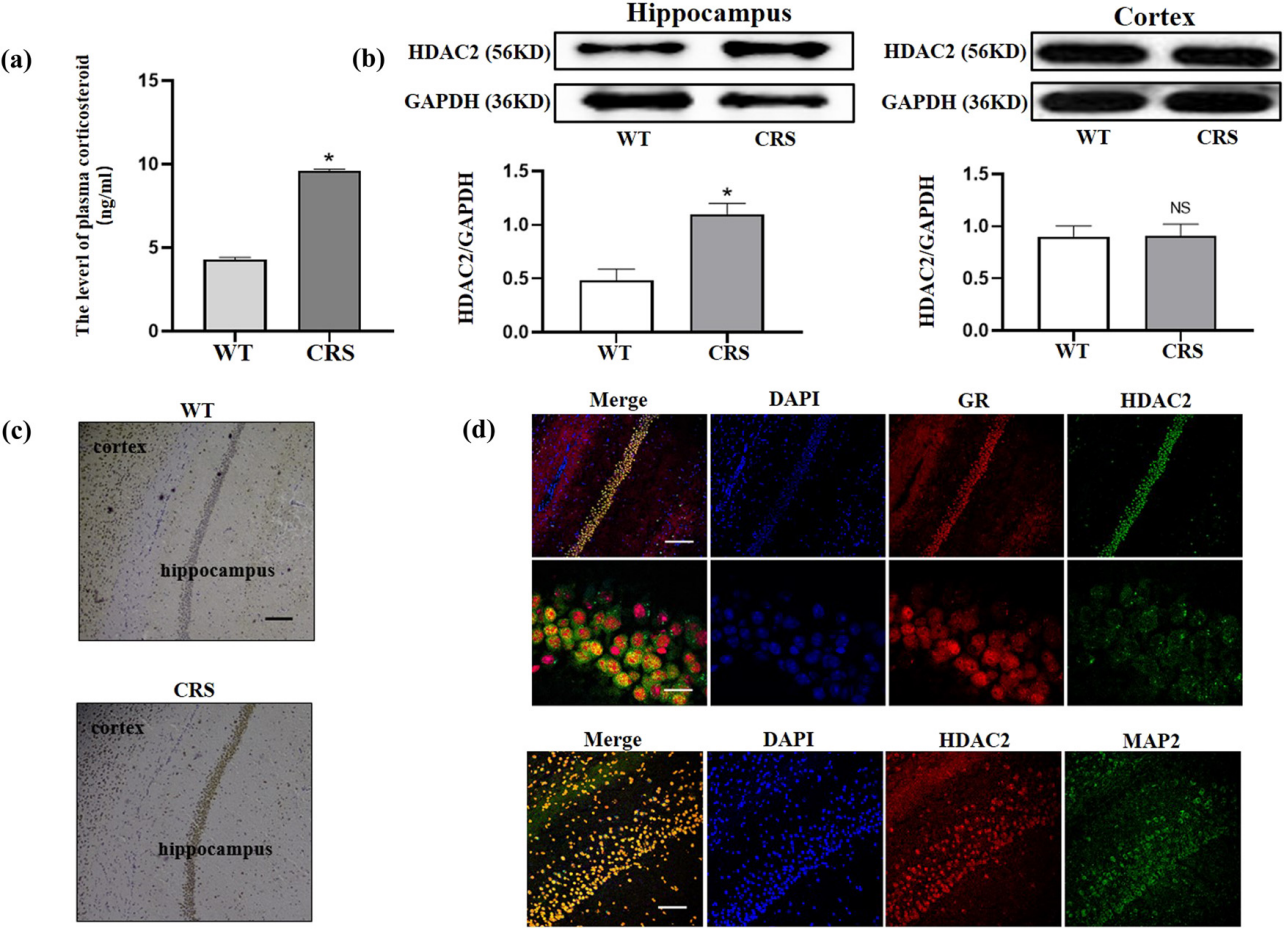
HDAC2 was increased in hippocampal neurons and was co-localized with GR following chronic restraint stress. (a) The plasma corticosteroid level was increased following chronic stress, *N* = 6, *p = 0.024. (b) The HDAC2 level after stress was upregulated in the hippocampus but not in the cortex, *N* = 3, *p = 0.0325; NS, not significant. (c) Immunohistochemical staining showed the increasing trend of HDAC2 in hippocampus after stress (bar = 100 μm). (d) The co-location of HDAC2 and GR as well as HDAC2 and MAP2 in hippocampus and cortex (bar = 100 μm).

Chronic stress mainly induces damage by upregulating glucocorticoid level. We speculated that glucocorticoids could pass through the blood–brain barrier (BBB) and enter the brain to impair cognitive functions. ELISA was used to detect plasma glucocorticoid levels in mice after chronic stress treatment (*p* = 0.023, Figure 3a) and showed an apparent increase in glucocorticoid level in chronic stress-treated mice compared to the control group.

### 3.4 HDAC2 was increased in hippocampal neurons and was co-localized with glucocorticoid receptors following chronic restraint stress

Western blot analysis of the cortex and hippocampus for total protein levels indicated that the hippocampal HDAC2 protein level was increased following chronic stress (*p* = 0.041), but that the cortical HDAC2 level did not change (Figure 3b). Immunohistochemical staining of brain sections showed that the hippocampal HDAC2 level in animals under chronic stress was much higher than the control mice (Figure 3c). To reveal the relationship between HDAC2 and glucocorticoid receptors (GRs) in hippocampal neurons, we performed immunofluorescence and found that HDAC2 was co-localized with GRs and the neuronal marker MAP2 (Figure 3d and e). These results suggest that increased HDAC2 is related to GR-mediated signaling pathways during chronic stress.

### 3.5 Chronic restraint decreased histone acetylation level in hippocampal neurons

**Figure 4:**
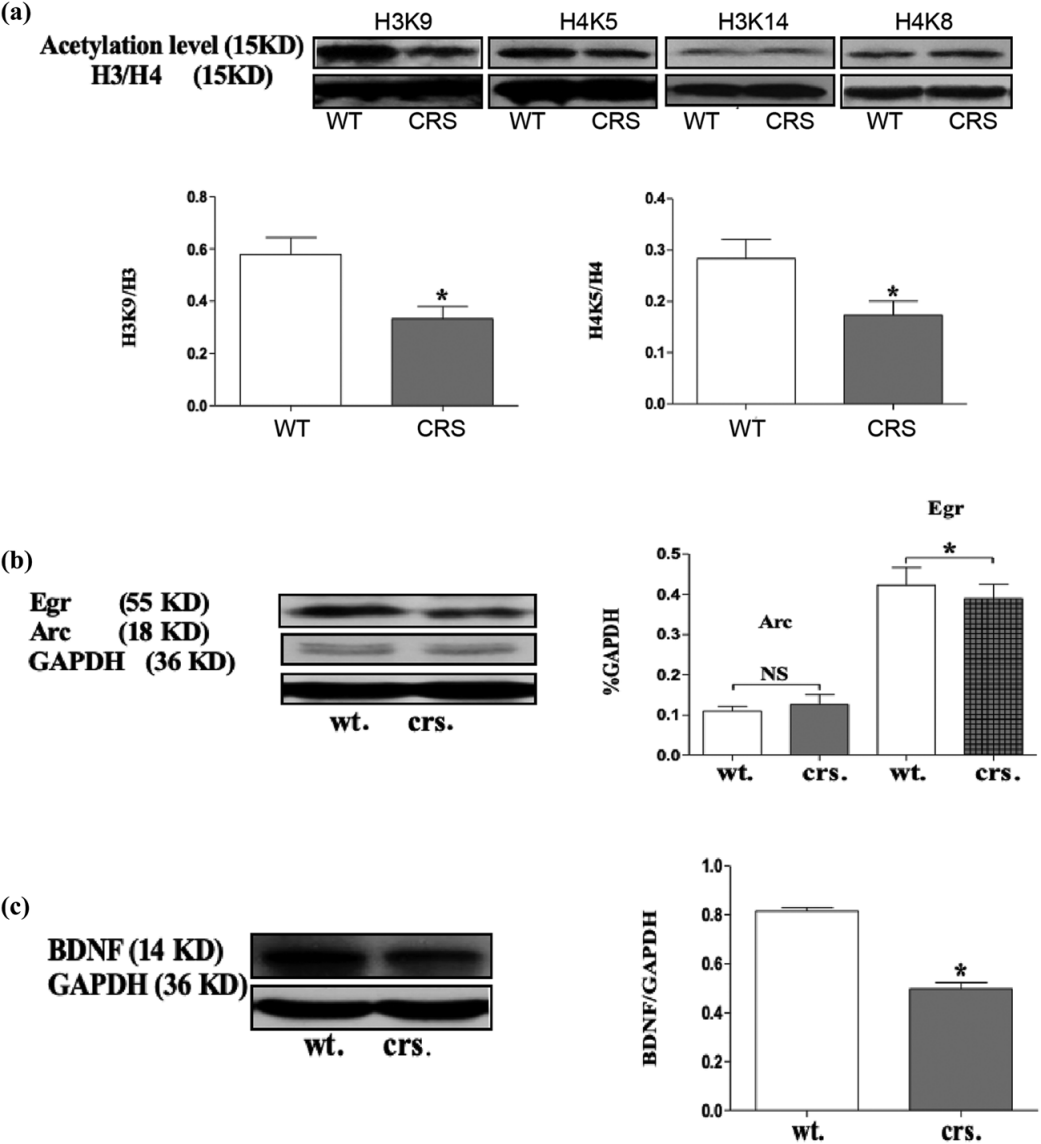
Chronic restraint decreased histone acetylation, IEGs and BDNF in hippocampal neurons. (a) Chronic stress decreased histones acetylation levels in the hippocampus H3K9: *p = 0.0480; H4K5: *p = 0.0410. (b) Chronic stress induced the downregulation of IEGs in the hippocampus, *p = 0.0490. (c) Chronic stress induced the downregulation of BDNF in the hippocampus, *p = 0.0481. *N* = 3.

Previous studies have reported that chronic stress or glucocorticoid release can reduce the level of histone acetylation and regulate the expression of related target genes [18]. We examined the acetylation level of several histone sites associated with cognition and, using western blot, showed that the levels of acetylation on H3K9 (*p* = 0.033) and H4K5 (*p* = 0.030) proteins were significantly decreased in mice with chronic stress (Figure 4a).

### 3.6 Chronic stress-induced downregulation of immediate early genes and BDNF

Compared with HDAC2-dependent gene regulation, immediate early genes (IEGs) can be rapidly activated by various factors in the early stages of stress [19,20]. In the nervous system, IEGs regulate the expression of many transcription factors, which are secreted proteins that are necessary for regulating cognitive functions [21]. Previous findings revealed that chronic stress can regulate some IEGs such as Arc and Egr. Therefore, here, we examined the expression of Arc and Egr and found that Egr (*p* = 0.042) and BDNF (*p* = 0.029) were significantly decreased and that Arc was not significantly changed in the hippocampus of mice under chronic stress (*p* = 0.058, Figure 4b and c).

### 3.7 Chronic stress inhibited the PI3K/AKT signaling pathway

**Figure 5:**
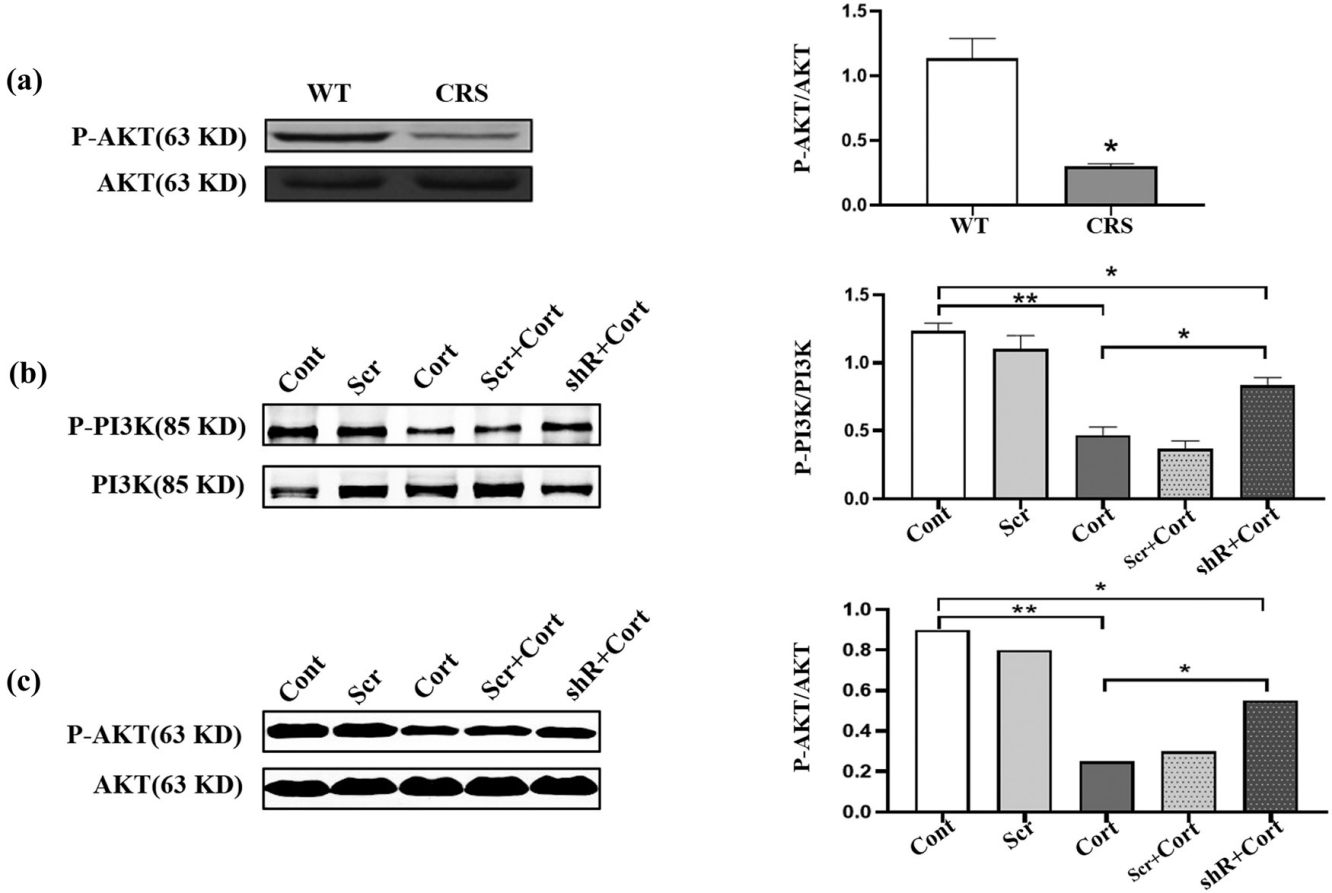
Chronic stress inhibited the PI3K/AKT signaling pathway. (a) Chronic stress induced the downregulation of AKT phosphorylation, *p = 0.013. (b) Corticosteroid (Cort) treatment decreased PI3 phosphorylation but HDAC shRNA reversed this effect in N2a cells. **p = 0.096; cont vs shr + cort: *p = 0.0152; cort vs shr + cort: *p = 0.0200. (c) Cort treatment decreased AKT phosphorylation but HDAC shRNA reversed this effect in N2a cells. **p = 0.087; cont vs shr + cort: *p = 0.0121; cort vs shr + cort: *p = 0.0230. *N* = 3.

Chronic stress often induces abnormal energy metabolism and the PI3K/AKT signaling pathway acts as an important part in energy metabolism regulation [22]. To further reveal the relation of a dysfunctional metabolism and memory impairment, we detected AKT phosphorylation in the hippocampus of mice (*p* = 0.022, Figure 5a). The results indicated that the phosphorylation level of AKT Ser473 was significantly decreased in mice experiencing chronic stress. To further explore the effects of stress on the PI3K/AKT signaling pathway, we used corticosteroid treatment on N2a cells to mimic the chronic stress environment. We observed that the phosphorylation of PI3 and AKT were reduced following corticosteroid treatment (*p* = 0.008). However, when HDAC2 expression was curbed with shRNA, those effects were reversed (*p* = 0.021) (Figure 5b and c). These results suggested that the HDAC2 is involved in the phosphorylation of PI3K-Akt signaling and mediate neuroprotective functions.

## 4 Discussion

In the present study, we found that chronic stress can induce significant cognitive impairment in mice. We further concluded that HDAC2 is involved in memory-associated gene expression that includes IEGs and BDNF. Cognitive impairment induced by chronic stress refers to the long-term exposure of the body to negative stimuli with intolerance, eventually causing impairment of learning and memory. Under chronic stress, glucocorticoid levels significantly increase, followed by the binding and activation of GRs, resulting in changes in multiple signaling pathways [23].

Corticosterone impairs cognitive functions via different pathways and mechanisms, including causing a reduction in histone acetylation, impeding the expression of related proteins, and affecting the functions of different biological functions [24]. Our results show that stress can significantly increase plasma glucocorticoid levels in mice. In the hippocampus and cortex of mice, GRs and HDAC2 are co-localized in neurons which suggest that they likely interact with one another. In chronic stress, a large number of glucocorticoids can enter the brain through the BBB, bind to the GR, and activate the glucocorticoid signaling pathways to affect HDAC2 expression [1,9,25]. Consistent with current speculation, we found that the level of HDAC2 in the hippocampus was significantly upregulated, suggesting that HDAC2 may be involved in the memory impairment induced by chronic stress.

The HDAC family is responsible for catalyzing histone deacetylation, and many specific HDAC family members, including HDAC2, have been identified to be involved in learning and memory regulation. HDAC2 can catalyze the deacetylation of certain histone lysine sites that are closely related to cognitive function, such as H3K4, H3K9, H4K5, and H4K12 [26]. HDAC2 belongs to class I of the HDAC family and is mainly located in the nucleus of cells [27]. It is widely expressed in the central nervous system and particularly abundant in cognition-related brain areas such as the cortex and hippocampus. Furthermore, many studies have reported that HDAC2 is particularly responsible for the negative regulation of cognitive function. In the mouse model of HDAC2 overexpression, HDAC2 negatively regulates synaptic plasticity and structural stability, inhibiting the formation of learning and memory. Moreover, *in vitro* and *in vivo* studies have confirmed that the HDAC inhibitor valproate can significantly improve LTP in neurons and improve cognitive functions.

In many neurogenerative diseases with cognitive dysfunction, HDAC2 is found to be upregulated, and HDAC2 inhibition or conditional knockout can significantly improve the learning and memory ability in affected mice [26]. In APP/PS1 mice, an AD mouse model, GRs can bind to the HDAC2 promoter region and result in increased levels of the HDAC2 protein in the hippocampus and subsequent activation of stress-related pathways. These results suggest that HDAC2 may participate in the process of cognitive dysfunction.

In this study, we found that the levels of H3K9 and H4K5 acetylation significantly decrease with the increase of HDAC2 in chronic stress [27]. Decreased histone acetylation impedes the expression of its target proteins which are associated with synaptic functions and include PSD95, GluR-1, GABAAR, Syn, and the neurotrophic factor BDNF [28]. PSD95 is located in the dense post-synaptic region and plays an important role in facilitating the anchoring and synaptic function of other proteins. PSD95 knockout impairs the spatial learning and memory in mice. GluR-1 is a glutamyl receptor that is expressed in synapses and GluR-1 knockout mice show apparent depression-like behaviors. BDNF is one of the most important neurotrophic factors and plays an important role in maintaining LTP, learning and memory formation in the hippocampus [29]. Our findings reveal that chronic stress reduces the expression of cognition-associated proteins and impairs cognitive ability by upregulating HDAC2 and decreasing the acetylation of H3K9 and H4K5. Furthermore, we observed changes to the IEGs Arc and Egr which can also influence gene expression in the early stage of stress. In conclusion, under chronic stress, gene expression can be regulated either via a slow response such as HDAC2-dependent deacetylation or by IEG-dependent regulation such as Arc and Egr.

Chronic stress often induces dysregulated energy metabolism but the specific mechanism needs further study. In our previous study, we found that the stress hormone orticosteroid upregulated HDAC2 protein levels in neuro-2a cells and caused cell injuries [30]. In the present study, we further confirmed that corticosteroid release could inhibit the phosphorylation of AKT and influence the functions of the PI3K/AKT signaling pathway [31]. The results revealed that increased levels of glucocorticoids could influence energy metabolism, and that an abnormal energy metabolism may be an important cause of cognitive impairment under chronic stress.

In conclusion, in our present study, chronic stress increased HDAC2 expression, mediating cognitive impairment in mice. Further studies are needed to elucidate the mechanism of HDAC2 upregulation under stress, which may provide new insights for HDAC2 regulation and assist in the development new drug targets for HDAC2 inhibitors.
